# ELEVATING HEALTHY AGING: HOW PUBLIC HEALTH IS SUPPORTING OLDER ADULTS

**DOI:** 10.1093/geroni/igac059.720

**Published:** 2022-12-20

**Authors:** Karon Phillips, Megan Wolfe

**Affiliations:** Trust for America's Health, Silver Spring, Maryland, United States; Trust for America's Health, Washington, District of Columbia, United States

## Abstract

Trust for America’s Health (TFAH) leads the national Age-Friendly Public Health Systems initiative which focuses on expanding the public health role in healthy aging. The roles of public health include creating and leading policy, systems, and environmental changes; connecting and convening multi-sector stakeholders to improve older adult health and well-being; and collecting and translating data on older adult health to inform interventions in communities and states. TFAH will share examples of how public health departments are elevating healthy aging as a core function.

